# Soft hydrogen bonds to alkenes: the methanol–ethene prototype under experimental and theoretical scrutiny

**DOI:** 10.1039/c5sc01002k

**Published:** 2015-05-11

**Authors:** Matthias Heger, Ricardo A. Mata, Martin A. Suhm

**Affiliations:** a Institut für Physikalische Chemie , Universität Göttingen , Tammannstr. 6 , 37077 Göttingen , Germany . Email: msuhm@gwdg.de

## Abstract

An FTIR spectroscopic study of the elusive hydrogen-bonded methanol–ethene complex, the most elementary example for weak intermolecular alcohol hydrogen bonding to a π cloud, is presented. By isolating the complex in a supersonic jet, the rigorous comparability to high-level quantum chemical calculations is ensured. In stark contrast to classical hydrogen bonds, experimental overtone analysis reveals the harmonic oscillator approximation for the OH red shift to be accurate. Harmonic calculations up to explicitly correlated local coupled-cluster level are thus found to agree very well with experiment. The experimental OH values for the red shift (45 cm^–1^), the small change in diagonal anharmonicity (–3 cm^–1^) and the overtone intensity attenuation (2 × 10^2^-fold) together with theoretical predictions for the preferred structural arrangement and the zero-point-corrected dissociation energy (8 kJ mol^–1^) may thus be regarded as definitive reference values for related systems and for more approximate computational methods. In particular, MP2 calculations are shown to fail for this kind of weak intermolecular interaction.

## Abstract 2

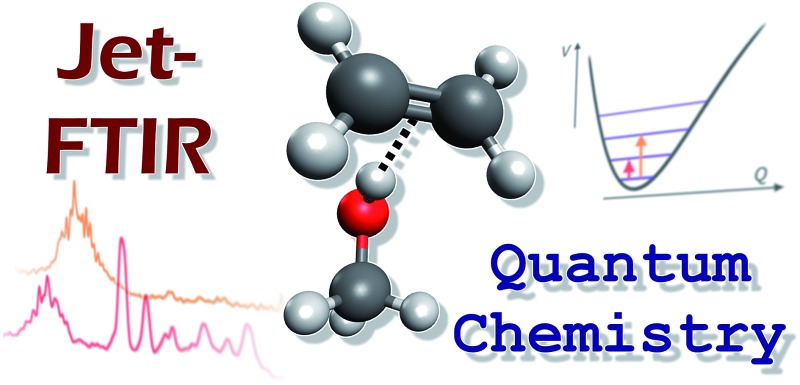
Theory meets experiment for the simplest model of alcohol–alkene hydrogen bonding and both support a close to harmonic description.

## 1 Introduction

Hydrogen bonds are ubiquitous in nature, governing molecular conformations and thus biochemical functionality. This holds not only for strong hydrogen bonds to heteroatoms, but also for weak OH···π interactions which have been associated with olfactory processes.^1^,^2^ Detection of such weak interactions typically relies on the study of the sensitive vibrational signature of the donor OH bond and the spectroscopic “red shift” that usually accompanies bond formation. However, the low interaction energies in (weak) hydrogen bonds complicate the experiments in that association of the molecular constituents is fleeting even at low temperatures. This demands for some sort of stabilization of the metastable clusters which is typically realized by means of supersonic expansions^3^ or cryogenic matrices.^4^ Embedding effects in the latter can distort the vibrational signature of weak OH···π bonds. Fixation of the donor and acceptor moieties in a common molecular frame is an alternative to increase the fraction of hydrogen-bonded structures, and O–H stretching red shifts of up to 90 cm^–1^ have been found for various such intramolecular OH···π interactions in different environments.^5^–^8^ However, it is difficult to elucidate the role that geometric strain and substituent effects have on the spectroscopic signatures in these cases. In contrast to aromatic OH complexes,^9^–^12^ unconstrained olefinic alcohol contacts remain surprisingly unexplored.

Quantum chemical calculations are customarily used to suggest structural motifs, dissociation energies and assignments to observed spectral features. Direct comparison between theory and experiment is typically hampered by the fact that anharmonic vibrational treatments are challenging except for small systems and rather simple methods. In addition, an experimental determination of anharmonicity *via* overtone bands^13^,^14^ suffers from their low infrared intensity, which decreases as the strength of the hydrogen bond increases (conversely to the fundamental band).^15^,^16^ Recently, we have been able to disentangle the 111 cm^–1^ red shift in the prototypical methanol dimer into its harmonic and anharmonic contributions, and high-level quantum chemical calculations have shown that many popular theoretical methods are inadequate for a quantitative description of the harmonic component.^17^,^18^ In combination with FIR data, a consistent picture has emerged in which the increase in diagonal anharmonicity of the OH stretching oscillator upon bond formation is overcompensated by a large coupling to OH-librational motion out of the bond, an effect which is absent in the free monomer.^18^ Both the diagonal and off-diagonal anharmonic effects depend on the strength of the hydrogen bond itself, and it will be most interesting to contrast this model OH···O bond with a prototypical weak OH···π bond.

**Fig. 1:**
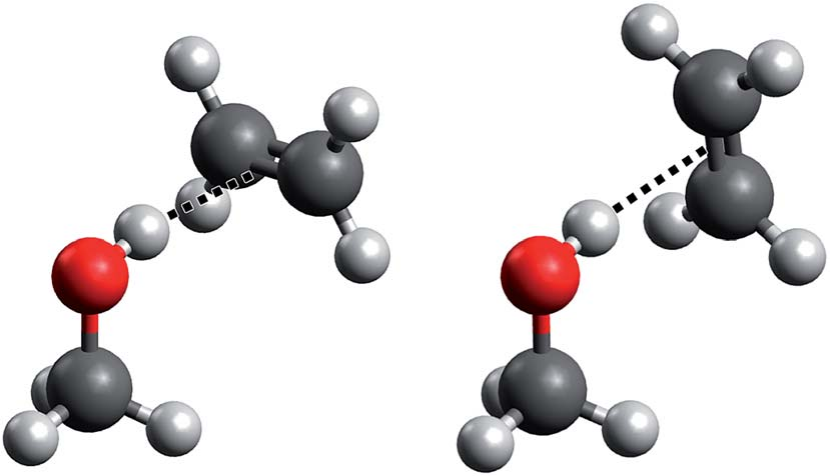
“Perpendicular” (left) and “parallel” (right) ME structures. Only the former is predicted to be a stable minimum, but low barriers to the torsion of the ethene unit may leave artifacts in harmonic and anharmonic approaches.

Such a prototypical hydrogen bond is found in the methanol–ethene model system, but it has so far only seen theoretical treatment in one study^19^ and no explicit experimental characterization whatsoever. Here, we present for the first time spectroscopic data on this important system (which we abbreviate “ME”), backed by high-level quantum chemical calculations. Further, the impact of the weak hydrogen bond on the anharmonicity of the OH stretching oscillator is characterized by both experiment and theory. We largely follow our earlier approach to the methanol dimer (“MM”)^17^,^18^,^20^ for which we also present new results from quantum chemical treatments. A specific problem that arises in methanol–ethene is the rotation of the ethene molecule around the hydrogen bond. In the equilibrium structure of the complex, the C=C bond is perpendicular to the mirror plane of the methanol molecule (see Fig. 1, left), as it has already been suggested previously.^19^ We confirm that the rotation of the ethene unit around the hydrogen bond exhibits almost no barrier, which becomes problematic in the global energy minimum predictions among many quantum chemical treatments. We will address this aspect in greater detail when discussing its impact on anharmonic VPT2 calculations.

## 2 Methods

### 2.1 Jet-FTIR experiment

The jet-FTIR experiments were carried out using the “filet” jet, which has been described in detail elsewhere.^21^ Its unmatched eponymous feature is the “*fi*ne, but *le*ng*t*hy” 600 × 0.2 mm^2^ slit nozzle which is fed by 6 solenoid valves from a 67 L Teflon-coated reservoir at typical stagnation pressures of *P*_S_ = 0.75 bar. The jet chamber is backed by 23 m^3^ of buffer volumes and pumped continuously at 2500 m^3^ h^–1^ pumping speed. The molecular beam is sampled by the mildly focused beam of a Bruker IFS 66v/S FTIR spectrometer at 2 cm^–1^ resolution, employing a 150 W tungsten lamp as the light source and CaF_2_ optics. Cooled InSb and InGaAs detectors are used for fundamental and overtone measurements, respectively, in conjunction with appropriate optical filters to narrow their bandwidths. Typically, spectra are averaged from about 50 to 100 single scans for the fundamental region and about 1000 scans for overtones. Sample preparation is carried out from thermostatted liquid methanol (“M”, Roth, ≥ 99.9%) through which a stream of helium is directed, and by admixture of ethene (“E”, Linde, 99.9%) in helium stored in a gas cylinder at 50 bar.

### 2.2 Quantum chemical methods

Quantum chemical calculations were carried out using the MOLPRO 2012.1 (ref. 22) and GAUSSIAN09 (ref. 23) software packages. The former features implementations of local-correlation methods (prefix “L”) which are advantageous in terms of computational resources while at the same time largely eliminating the basis set superposition error,^24^ providing robust harmonic frequencies.^25^ Specifically, we rely on the explicitly correlated LCCSD(T*)-F12a method^26^,^27^ with scaled triples (“T*”). The F12a ansatz was chosen over F12b for its fortuitous error cancellation observed when used in combination with small basis sets.^28^ Inclusion of all intermolecular orbital pairs in the coupled-cluster correlation treatment, which is mandatory for correct predictions, is indicated by a suffix “(int)”.

GAUSSIAN09 was used for canonical MP2 and B2PLYP-D3BJ calculations (including Grimme's empirical D3 dispersion^29^ and Becke–Johnson damping^30^). Further, anharmonic VPT2 calculations as implemented in the software package^31^,^32^ were carried out in order to obtain explicit estimates of anharmonicity constants, using the int = ultrafine grid integration option at the DFT level.

Most *ab initio* calculations were done using Dunning's correlation-consistent basis sets (aug-)cc-pV*n*Z,^33^,^34^ which we abbreviate “(a)V*n*Z”. For the explicitly correlated calculations, the VDZ-F12 basis set was used.^35^ The use of explicit correlation in combination with the latter basis should be enough to provide results comparable to quadruple-zeta calculations or better. Density fitting was employed throughout all local calculations, using the program's default aV*n*Z/JKFIT^36^ and aV*n*Z/MP2FIT^37^ basis sets; the F12a calculations made use of the VDZ-F12/OPTRI basis set.^38^

In the current study, we refer to the LCCSD(T*)-F12a(int)/VDZ-F12 method as our benchmark level of theory, as it has been found to be essentially converged to the basis set limit in the methanol dimer^17^ while being computationally feasible even for numerical gradient and Hessian calculations.

## 3 Results and discussion

### 3.1 Jet-FTIR spectra

**Fig. 2:**
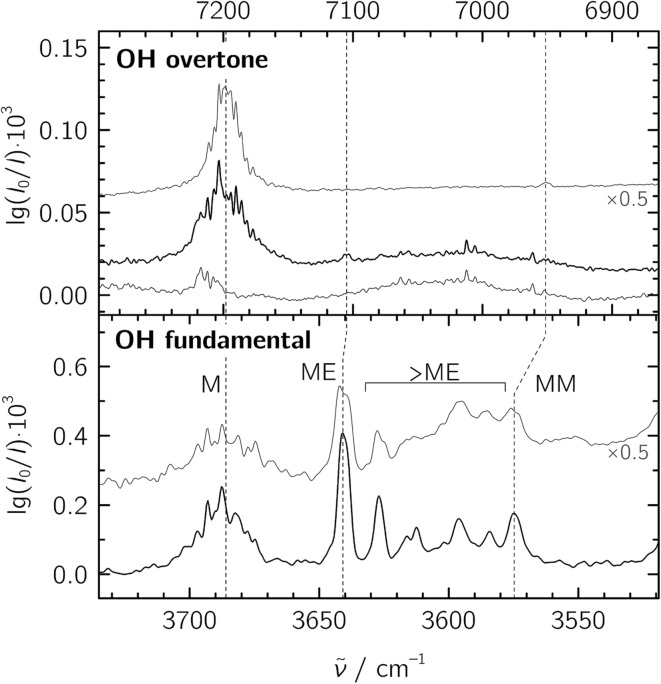
Jet-FTIR spectra of methanol : ethene mixtures in the fundamental (bottom) and overtone (top) regions. Bottom panel: mixtures with M : E ratios of ∼1 : 20 (black trace, with approximate number densities of 3 × 10^13^ cm^–3^ for M, 7 × 10^11^ cm^–3^ for MM and 3 × 10^12^ cm^–3^ for ME based on anharmonic B2PLYP-D3BJ/VTZ IR intensities) and ∼1 : 7 (grey trace, intensity-scaled by 0.5) expanded at a stagnation pressure of *P*_S_ = 0.75 bar. “>ME” indicates signals from larger clusters which we do not interpret explicitly. Top panel: overtone spectra of the ∼1 : 7 M : E mixture (strong black trace), E (thin black trace) and M (grey trace, from ref. 20, intensity-scaled by 0.5). The wavenumber scale in the top panel is compressed by a factor of 2 and shifted to match the M monomer band centers in order to visualize the change in diagonal anharmonicity in the ME and MM structures.

**Fig. 3:**
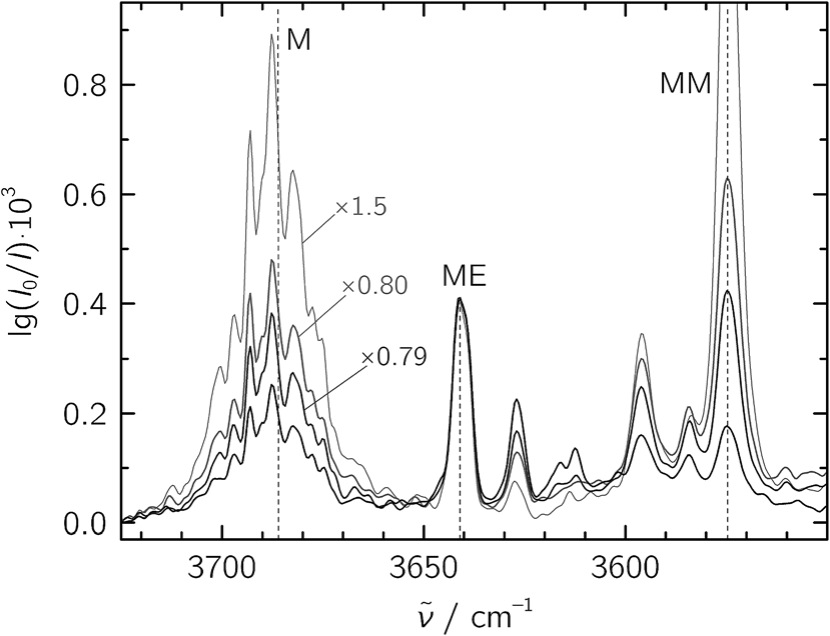
Jet-FTIR spectra of various M : E mixtures, with decreasing relative E concentration from strong to light traces. The strongest, black trace corresponds to the “∼1 : 20” spectrum shown in Fig. 2, with all other spectra scaled to its 3641 cm^–1^ ME band (scaling factors annotated).

For the jet-FTIR measurements in the fundamental region, a 2% mixture of ethene in helium was used, while the methanol concentration was controlled by cooling the liquid to –25 °C. Together with the opening and closing times of the solenoid valves feeding the reservoir, we estimate a M : E ratio of about 1 : 20 in a 1300-fold excess of He, which was expanded at a stagnation pressure of *P*_S_ = 0.75 bar. Lower stagnation pressures down to 0.40 bar were also used to decrease the amount of larger aggregates. We identify the mixed ME dimer band at 3641 cm^–1^ (see Fig. 2), which corresponds to a red shift of 45 cm^–1^ from the methanol monomer fundamental position at 3686 cm^–1^. The ME band is only 2 cm^–1^ higher in wavenumber than the corresponding band in the size-selected methanol–benzene complex,^11^ supporting our mixed dimer assignment. Further cluster bands arise at lower wavenumber (“>ME” in Fig. 2), which can be attributed to a bulk of ethene-rich structures and few distinct OH···OH stretching band pairs from methanol-rich clusters on grounds of their larger red shifts.^39^ This assignment is underscored by the distinct intensity evolution of these bands with respect to the 3641 cm^–1^ band when varying the relative ethene concentration (Fig. 3). A more detailed analysis of these larger structures is out of scope for the current study and will be revisited later.

To facilitate the overtone measurements, a higher ME abundance in the expansion was obtained by using a richer 10% ethene mixture at a higher reservoir feeding pressure of 1.8 bar while raising the methanol temperature to –15 °C, which results in a ∼1 : 7 M : E mixture in a 200-fold excess of He. This increases the rotational temperature and reveals the asymmetry of the ME band as being most likely due to residual rotational structure. Comparison of the fundamental and overtone spectra (Fig. 2) allows for the leading diagonal anharmonicity constant *x*_OH,OH_ of the OH oscillator to be extracted from the fundamental and first overtone band positions *ν̃*fundOH and *ν̃*otOH, respectively, with
1

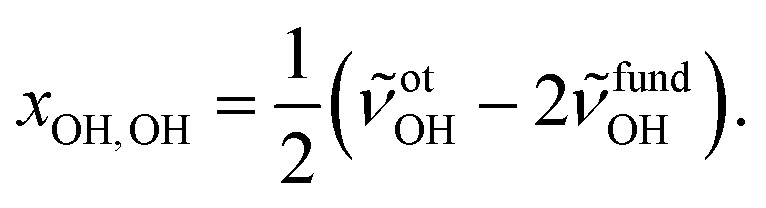

For the methanol monomer, *ν̃*fundOH = 3686 cm^–1^ and *ν̃*otOH = 7198 cm^–1^ yield a diagonal anharmonicity constant *x*_OH,OH_ of about –86 cm^–1^.^20^ This anharmonicity increases to –99 cm^–1^ upon formation of the OH···O hydrogen bond in MM with *ν̃*fundOH = 3575 cm^–1^ and *ν̃*otOH = 6951 cm^–1^.^20^ Applying the same analysis to the 7105 cm^–1^ ME overtone band position from our spectra, we deduce a diagonal anharmonicity constant *x*_OH,OH_ = –89 cm^–1^ for the dimer, which differs only slightly from the monomer value.

In addition, anharmonic cross-terms *x*_OH,i_ coupling the OH stretching motion to other vibrational modes must be considered when analyzing the fundamental band positions:
2

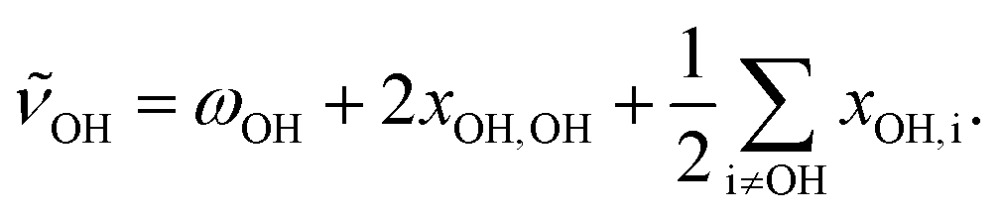

In the methanol monomer, the cross-terms *x*_OH,i_ were shown to be much smaller than 2*x*_OH,OH_.^20^ However, the low-barrier torsional motion of the OH group becomes hindered upon formation of the hydrogen bond, and a distinct positive librational coupling term *x*_OH,lib_ to the stretching mode arises; in the homodimer, it amounts to some 60 cm^–1^. This value is again sensitive to the strength of the hydrogen bond, but cannot be assessed from our spectroscopic data alone without observing weak combination or hot bands.

One further analysis involves the observed intensities of the fundamental and overtone bands, with the fund : ot ratio predicted to increase with stronger hydrogen bonds (*i.e.*, the first overtone to become weaker in comparison).^15^ In our experiment, the overtone intensity has to be down-scaled by a factor of 0.83(3) to account for the change in detectors with different areas between the two measurements. From the spectra of the rich M : E mixture, we find a fund : ot ratio of 170(70) which is significantly lower than the 320(90) ratio found for the MM homodimer.^20^

Overall, the small red shift, low increase in anharmonicity and modest overtone intensity attenuation bear witness to the weakness of this model OH···π hydrogen bond when compared to the MM case.

### 3.2 Harmonic wavenumbers and dissociation energies

As shown previously for the methanol dimer,^17^ advancing beyond the MP2 treatment of correlation allows to improve the predictions for the harmonic red shift –Δ*ω* of the donor O–H bond; this even holds when only this specific bond is selected for a higher correlation treatment. The latter is possible in the LMOMO scheme^40^ which allows to single out localized electron pairs to be treated by a different method than the remainder of the system. This approach has proven to resemble full LCCSD(T)(int) closely in MM with a strongly reduced cost for numerical gradient and Hessian calculations.^17^ For the donor O–H vibration, the benchmark LCCSD(T*)-F12a(int)/VDZ-F12 method predicts a harmonic red shift of –122 cm^–1^ which MP2/aVTZ and LMP2/aVTZ overestimate by 35 and 20%, respectively. It appears that popular MP2 approaches are at best qualitatively useful for analyzing the spectroscopic data for this important intermolecular contact. However, by applying Grimme's spin component scaling^41^ approach in SCS-LMP2/aVTZ, we find that the harmonic red shift is brought down to 113 cm^–1^, in much better agreement with our benchmark value. The electronic and harmonically zero-point corrected dissociation energies are then predicted some 2–3 kJ mol^–1^ too low, at *D*_e_ = 20.1 and *D*h0 = 15.1 kJ mol^–1^ as compared to 22.9 and 16.8 kJ mol^–1^ at the benchmark level; this suggests that the SCS harmonic shift performance also profits from error compensation.

For the ME dimer, the F12 calculations yield a harmonic red shift of –Δ*ω* = 45 cm^–1^. Again, canonical and local MP2 methods overshoot by some 33–56% (see Table 1) while SCS-LMP2/aVTZ provides a harmonic red shift that almost coincides with the benchmark data (see Table 1). For comparability with our previous MM study, we further present LMOMO calculations in which the methyl group and the adjacent C–O bond are reduced to an MP2 treatment while the rest of the system remains correlated at the CCSD(T)(int) level. We present them here only for the sake of completeness while encouraging the use of explicit correlation.

**Table 1:** Dissociation energies *D*_e_ and *D*h0, harmonic red shifts –Δ*ω* with deviations to the LCCSD(T*)-F12a(int)/VDZ-F12 benchmark in parentheses, and harmonic ethene-torsion wavenumbers *ω*_tors_ for the ME dimer on various levels of theory

	*D* h 0 (*D*_e_)/kJ mol^–1^	–Δ*ω*/cm^–1^	*ω*_tors_ ^*a*^/cm^–1^
B2PLYP-D3BJ/VTZ	11.0 (14.5)	54 (+20%)	17
MP2/VTZ	11.1 (14.6)	60 (+33%)	15
MP2/aVTZ	11.2 (14.7)	70 (+56%)	1
LMP2/aVTZ	8.5 (11.7)	64 (+42%)	9*i*
SCS-LMP2/aVTZ	6.3 (9.3)	43 (–4%)	7*i*
LMOMO/aVTZ^*b*^	6.7 (10.5)	39 (–13%)	13
LCCSD(T*)-F12a(int)/VDZ-F12	7.7 (10.9)	45	7

^*a*^Torsion of ethene around OH···π bond.

^*b*^LCCSD(T)(int): LMP2 LMOMO scheme; see text for details.

Overall, the weakness of the ME hydrogen bond becomes apparent from the ∼10 kJ mol^–1^ gap of the dissociation energies to the MM dimer with its best-estimate harmonic *D*h0 of 18.3 kJ mol^–1^.^17^

### 3.3 OH stretching anharmonicity

One important contribution to the overall experimental OH red shift in the methanol homodimer is the anharmonic cross-term that couples the stretching motion and the hindered rotation (libration) in the dimer. Since the latter motion tends to weaken the hydrogen bond, *x*_OH,lib_ has a positive sign, blue-shifting the stretching band from its diagonally anharmonic value. The effect on the librational motion itself was confirmed by means of matrix isolation spectra^18^ which lend credibility to the F12 benchmark harmonic and VPT2 results. In this light, the predicted harmonic red shift of Δ*ω* = –45 cm^–1^ and subtle change in diagonal anharmonicity of Δ*x*_OH,OH_ = –3 cm^–1^ in the ME system suggest that the stretching–libration coupling *x*_OH,lib_ is only on the order of ∼10 cm^–1^, much lower than in the homodimer (∼60 cm^–1^). While this is qualitatively expected for a weak OH···π bond, it represents an interesting case where the observable red shift can be explained to a good approximation by harmonic effects alone, given that diagonal and off-diagonal anharmonic contributions are small and mutually canceling. Together with the sensitive ethene torsion preference, the ME dimer thus provides a nice accuracy test for quantum chemical methods without the need to evaluate anharmonic effects.

Perturbational anharmonic treatments are available in the GAUSSIAN program package^31^,^32^ and have previously been applied to the methanol dimer.^18^ Predictions for the anharmonic terms *x*_OH,i_ of the donor OH stretching vibrations in MM and ME are given in Table 2. If the predicted anharmonic corrections are combined with benchmark LCCSD(T*)-F12a(int)/VDZ-F12 harmonic references, good agreement with the true experimental band positions is obtained. As expected, the stretching–libration coupling is markedly smaller in ME than in the homodimer and approximately cancels the diagonal anharmonic weakening of the stretching potential.

**Table 2:** Anharmonicity constants *x*_OH,i_ from VPT2 calculations (all using the VTZ basis set) for the methanol (donor) OH-stretching vibrations in M, MM and ME, together with the respective harmonic wavenumbers *ω*_OH_ and resulting anharmonic band positions *ν̃*_OH_. The primed sum over the cross-terms indicates exclusion of the stretching–libration coupling. Also given are estimates for *ν̃*_OH_ using benchmark LCCSD(T*)-F12a(int)/VDZ-F12 harmonic wavenumbers *ω*_OH_ of 3862, 3740 and 3817 cm^–1^ for M, MM and ME, respectively (“*ν̃*benchm.OH”). All data in cm^–1^

	M		MM		ME	
	B2PLYP-D3BJ	MP2	B2PLYP-D3BJ	MP2	B2PLYP-D3BJ	MP2
*ω*_OH_	3858	3882	3718	3740	3804	3823
*x*_OH,OH_	–86	–83	–103	–102	–91	–88
*x*_OH,lib_	+4	+9	+59	+59	+13	+17
∑′*x*_OH,i_^*a*^	–29	–30	+16	+10	–3	–4
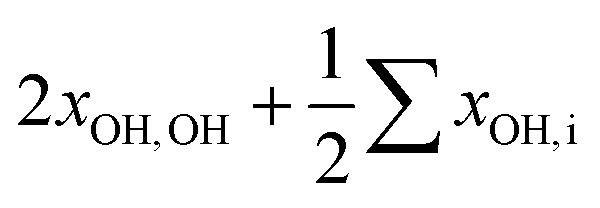	–185	–176	–168	–169	–177	–171
*ν̃*_OH_	3674	3706	3550	3571	3627	3652
*ν̃* benchm. OH ^*b*^	3677	3686	3572	3571	3641	3647
Experiment		3686		3575		3641

^*a*^Summed cross-terms, excluding *x*_OH,lib_.

^*b*^Using harmonic wavenumbers *ω*_OH_ at the LCCSD(T*)-F12a(int)/VDZ-F12 benchmark level.

Similar MP2/aVTZ calculations were conducted (not included in Table 2) which deviate markedly from these results, with an anharmonic ME band position of *ν̃*_OH_ = 3564 cm^–1^. Closer inspection reveals that this is in part due to the difficult ethene torsion which is predicted at a harmonic wavenumber of *ω*_tors_ ≈ 1 cm^–1^. While the corresponding stretching-ethene torsion coupling term *x*_OH,E-tors_ amounts to about 0.5 and –0.03 cm^–1^ in the robust B2PLYP-D3BJ/VTZ and MP2/VTZ calculations, respectively, it is –98 cm^–1^ at this faulty level of theory. We attribute this to a BSSE effect caused by diffuse functions on the hydrogen atoms. When neglecting this error, the overall MP2/aVTZ anharmonic correction is about –177 cm^–1^, in agreement with the robust calculations (see Table 2); however, the summed cross-terms, barring the OH libration, amount to –13 cm^–1^ as compared to –3 to –4 cm^–1^. While not drastic, this deviation cautions against taking contaminated VPT2 results out of context even if the error source can be identified.

The VPT2 calculations further provide anharmonic infrared intensities for the fundamental and overtone bands under scrutiny. From MP2/VTZ and B2PLYP-D3BJ/VTZ, we find predicted fund : ot ratios of about 360 to 420 for MM and 150 to 170 for ME, respectively. The MM results are in adequate agreement with the experimental value of 320(90),^20^ while the ME results reproduce the experimental value of 170(70) very well. Despite the quite different character of these two model hydrogen bonds, perturbational treatments thus produce reasonable anharmonic estimates for the OH stretching mode, and combination with high-level harmonic reference wavenumbers brings them into good agreement with the absolute band positions observed in our experiments.

Estimating anharmonicity constants with our explicitly/locally correlated benchmark method is difficult due to the lack of a comparable implementation in the MOLPRO program package. We thus calculated potential energy curves along the (donor) O–H stretching normal modes *Q* in the methanol monomer and the two dimers. We fit a modified Morse potential of the form
3

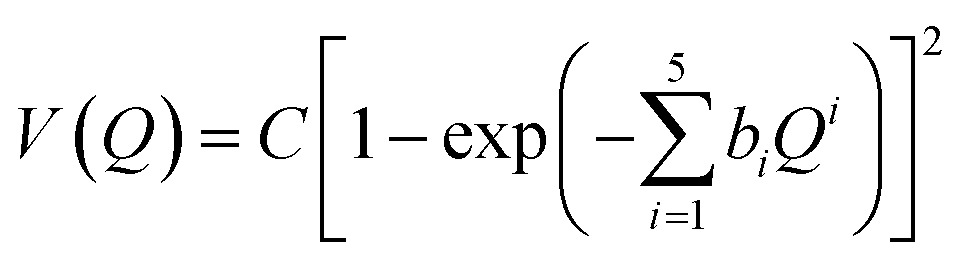

to the calculated energies, with *C* and *b*_1_ through *b*_5_ left free in the fit. We refrain from denoting the prefactor as a dissociation energy, since the resulting potential is not strictly dissociative anymore. Solutions to the vibrational Schrödinger equation were found by numerical variational calculations with a basis set of Gaussian functions distributed along the coordinate *Q*, using the reduced masses from the respective normal modes. The results are displayed in Table 3. The overtone is converged to below 10^–2^ cm^–1^ with respect to changes in number, spacing and width of the basis functions. Harmonic wavenumbers at the equilibrium position provide a consistency check with the normal-mode calculations, showing deviations up to 3 cm^–1^. We attribute these to fitting errors and assume the same variations for the calculated energy levels. Among our three test cases, the most interesting system is the methanol monomer, since the torsional perturbations of the OH oscillator – which cannot be captured with a 1-D model – are smallest there. The experimental wavenumbers are reproduced well by the benchmark method; conversely, the MM wavenumbers are underestimated due to the lack of this specific coupling. Still, the results are compatible with the blue-shifting *x*_OH,lib_ ≈ 60 cm^–1^ coupling suggested by the VPT2 calculations. Overall, the diagonal anharmonicities of the OH stretching oscillator from variational and VPT2 calculations show a satisfying agreement with the experiment across our methods even when the corresponding harmonic results are unreliable.

**Table 3:** Estimates of diagonal anharmonicity at the LCCSD(T*)-F12a(int)/VDZ-F12 benchmark level of theory, obtained from 1D variational calculations (“var.”) for the OH stretching oscillator in the methanol monomer (“M”) and the donor in the pure and mixed dimers (“MM”, “ME”). Also included are harmonic wavenumbers as a consistency check with normal-mode calculations (“norm.”). All data in cm^–1^

	*ω*_OH_		*ν̃* fund OH		*ν̃* ot OH		*x*_OH,OH_	
	Var.	Norm.	Var.	Exp.	Var.	Exp.	Var.	Exp.
M	3862	3862	3689	3686	7207	7198	–85	–86
MM	3737	3740	3547	3575	6902	6951	–96	–99
ME	3819	3817	3641	3641	7108	7104	–88	–89

As previously noted, the computed dissociation energies for the methanol–ethene system can be found in Table 1. However, in order to obtain quantitative estimates, the electronic energy should be recomputed with a larger basis set to converge the one-particle space. We carried out LCCSD(T*)-F12a(int)/VQZ-F12 single point calculations on the optimized VDZ-F12 structures, and obtained *D*_e_ = 11.4 kJ mol^–1^. This corresponds to a variation of only 0.5 kJ mol^–1^ when compared to the double-zeta result. It shows the good convergence of the value relative to the basis set. Given that these are all coupled cluster values, the error bar for *D*_e_ should be around 1 kJ mol^–1^. This is a rather conservative estimate. Adding the harmonic zero-point energy corrections, we obtain a value of *D*h0 = 8.2 kJ mol^–1^. In order to obtain a more reliable estimate of the spectroscopic dissociation energy, accurate anharmonic calculations for the zero-point energy would be required. However, these are extremely challenging given the large amplitude motions present in the system.

## 4 Conclusions

We have recorded FTIR spectra of methanol : ethene mixtures in supersonic expansions, assigning the fundamental and overtone transitions of the mixed dimer. The observed OH stretching red shift –Δ*ν̃*_OH_ = 45 cm^–1^ from the monomer reference is reduced by about 60% from that of the homodimer. The weakness of this prototypical OH···π contact is further attested by the minute change in diagonal anharmonicity of Δ*x*_OH,OH_ ≈ –3 cm^–1^ and moderate 170(70)-fold intensity attenuation of the overtone with respect to the fundamental.

High-level quantum chemical calculations with local and explicit electron correlation treatment predict a harmonic red shift of –Δ*ω*_OH_ = 45 cm^–1^ which coincides with the experimental anharmonic value. Assuming the chosen method to be robust, the observed wavenumber shift is thus mostly a harmonic effect, indicating that diagonal and off-diagonal anharmonic corrections closely cancel each other. As in the methanol homodimer,^20^ the most important contributions come from the diagonal term of the OH stretching vibration and the off-diagonal stretching–libration coupling; in the methanol–ethene dimer, the latter is predicted by VPT2 calculations at only 13–17 cm^–1^, providing another measure for the weakly perturbing character of the intermolecular interaction. Likewise, the harmonic zero-point dissociation energy at our best level of theory is *D*h0 = 8.2 kJ mol^–1^, 55% less than in the methanol dimer (*D*h0 = 18.3 kJ mol^–1^).^17^ Allowing for possible anharmonic effects in both directions for this floppy system, a conservative estimate of 8.2 ± 2.0 kJ mol^–1^ for the spectroscopic dissociation energy of ME appears justified. Microwave verification of the subtle structural preference of the methanol–ethene complex for a perpendicular arrangement of the C–O and C=C axes would be welcome.

We reiterate our previous findings that the MP2 method is inadequate for harmonic wavenumber predictions in alcoholic hydrogen bonds, significantly overestimating the red shift in canonical and local correlation treatments. However, SCS-LMP2 fares well in this regard both for the weak OH···π methanol–ethene and stronger OH···O methanol–methanol contacts, at the well-known^42^ expense of underestimating the dissociation energy. The quantitative insights into OH···π interactions obtained for methanol–ethene can help to advance our understanding of pre-reaction complexes in olefin epoxidation,^43^ hydroxyl radical reactions,^44^ electric field effects in OH···π contacts^12^ and the subtle donor–acceptor balance in methanol–ethyne.^45^
